# Effect of Incentive Spirometry Exercise on Perceived Stress in Chronic Obstructive Pulmonary Disease Patients: A Quasi‐Experimental Study

**DOI:** 10.1002/hsr2.72408

**Published:** 2026-04-17

**Authors:** Noor Nasir Rajpoot, Noman Sadiq, Quang Dai La, Tahira Sadiq, Eric Teng, Han B. La, David M. Lo

**Affiliations:** ^1^ Department of Medicine Mekran Medical College Turbat Pakistan; ^2^ Department of Physiology Mekran Medical College Turbat Pakistan; ^3^ Department of Biology Texas A&M University College Station Texas USA; ^4^ Ministry of National Guard Hospital Riyadh Saudi Arabia; ^5^ Department of Public Health Texas A&M University College Station Texas USA; ^6^ Department of Medicine Texas A&M Naresh K. Vashisht College of Medicine Bryan Texas USA; ^7^ Department of Biology Rutgers University New Brunswick New Jersey USA; ^8^ Department of Family Medicine Garnet Health Medical Center Orange County New York USA

**Keywords:** breathing exercises, chronic obstructive pulmonary disease (COPD), exercise therapy, incentive spirometry, perceived stress, psychological health, pulmonary rehabilitation, quality of life, quasi‐experimental study, stress reduction

## Abstract

**Background and Aims:**

Patients with chronic obstructive pulmonary disease (COPD) can have a decrease in quality of life because of both psychosocial and physical restrictions. Breathing exercises, such as incentive spirometry (IS), can be beneficial for physical outcomes; however, less is known about the effectiveness of IS on stress, which is explored in this study.

**Methods:**

This quasi‐experimental pretest‐posttest study was conducted for 1 year after getting ethical approval. One hundred participants diagnosed with grade 0–2 COPD according to MMRC grading were recruited and were assigned to either an exercise group or a control group using a convenient purposive sampling technique. Patients completed a perceived stress questionnaire at baseline and after 8 weeks of IS intervention. Statistical analyses were completed with independent and paired sample *t*‐tests.

**Results:**

Ninety‐two patients completed the study. Baseline perceived stress scores did not differ significantly across groups, *p* > 0.05. After 8 weeks, the IS perceived stress completion scores significantly decreased compared to the control, *p* < 0.05. Control group perceived stress scores showed no statistically significant changes, *p* > 0.05.

**Conclusion:**

Incentive spirometry provides a reliable non‐pharmacological intervention to alleviate perceived stress in patients with COPD. IS can be useful as part of a COPD rehabilitation program, not only for physical outcomes, but also for perceived psychological benefits.

## 1 Introduction

**Figure 1:**
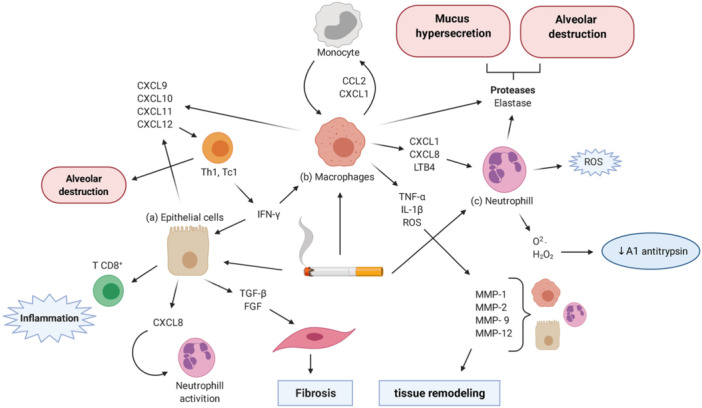
An outline of the molecular and cellular processes that underlie the pathophysiology of COPD. Toxins from cigarette smoke stimulate macrophages, epithelial cells, and lymphocytes, which causes chemokines and cytokines to be released. These cell types attract neutrophils, monocytes, fibroblasts, and CD8 T cells. In contrast to macrophages, which release pro‐inflammatory mediators (TNF‐α, IL‐1β) that promote matrix metalloproteinase (MMP) activity and airway remodeling, neutrophils cause tissue damage by producing reactive oxygen species (ROS) and proteases. By secreting chemokines (CXCL8, CXCL9–12) and growth factors (TGF‐β, FGF) that encourage fibrosis, epithelial cells further intensify inflammation. These mechanisms work together to cause the structural alterations, alveolar damage, and persistent inflammation that are hallmarks of COPD. CCL, C‐C motif chemokine ligand; CXCL, C‐X‐C motif chemokine ligand; IFN‐γ, interferon gamma; IL, interleukin; LTB4, leukotriene B4; MMP, metalloproteinase; NE, neutrophil elastase; ROS, reactive oxygen species; TNF‐α, tumor necrosis factor alpha. *Source:* Image reproduced from Rodrigues et al., 2021, created with BioRender.com [3]. Reproduced with open access permissions under the terms and conditions of the Creative Commons Attribution (CC BY) license (https://creativecommons.org/licenses/by/4.0/).

Chronic Obstructive Pulmonary Disease (COPD) is a chronic respiratory condition characterized by airflow obstruction, persistent cough, and progressive shortness of breath, which is not completely reversible and often leads to repeated exacerbations [1, 2]. The development and progression of COPD can be caused by ROS, inflammatory mediators, and the generation of proteolytic enzymes, which can start, intensify, and worsen tissue damage and lung injury (Figure 1) [3]. It encompasses emphysema and chronic bronchitis [2, 4]. COPD contributes substantially to morbidity and mortality through functional impairment, reduced social participation, and frequent hospitalizations [5, 6, 7]. Globally, more than 210 million people are affected, with tobacco smoking being the leading risk factor, though environmental exposures, occupational hazards, and childhood respiratory infections are also contributors [8, 9, 10]. In Pakistan, 36% of men and 9% of women consume tobacco daily, resulting in a COPD prevalence of 11.31% [11, 12].

**Figure 2:**
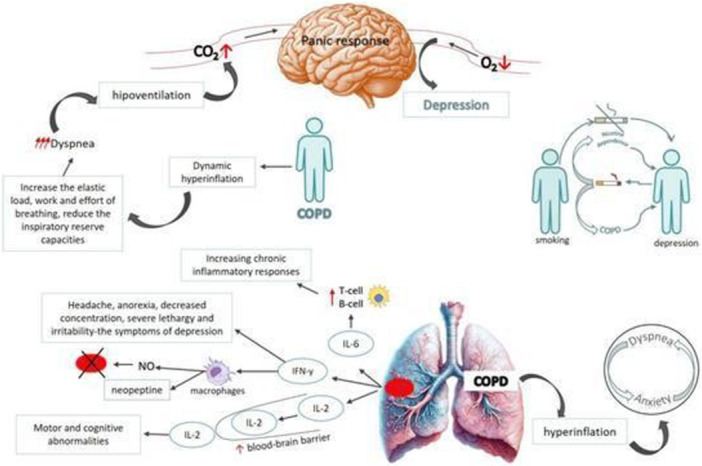
Proposed mechanisms linking chronic obstructive pulmonary disease (COPD) and depression. Shared pathways include systemic and neuroinflammation (IL‐6, IFN‐γ, IL‐2), hypoventilation, hypercapnia, and hypoxia, which contribute to cognitive dysfunction and depressive symptoms. Dyspnea and dynamic hyperinflation intensify anxiety, while nicotine dependence perpetuates a cycle connecting smoking, depression, and COPD. B‐cell, B lymphocyte; CO₂, carbon dioxide; IFN‐γ, interferon gamma; IL, interleukin; NO, nitric oxide; O₂, oxygen; T‐cell, T lymphocyte. *Source:* Image reproduced from Corlateanu et al., 2025 [13]. Reproduced with open access permissions under the terms and conditions of the Creative Commons Attribution (CC BY) license (https://creativecommons.org/licenses/by/4.0/).

In addition to physical disability, COPD has profound psychological consequences (Figure 2) [13, 14]. Studies indicate that depression, anxiety, and stress occur in 12%–50% of COPD patients, compared with about 5% in the general population [15]. These psychological comorbidities interfere with disease management, reduce adherence to smoking cessation programs, and increase the risk of hospitalization [16]. The Perceived Stress Scale (PSS), a validated 10‐item tool, is widely used to assess stress levels in clinical populations [17, 18, 19].

Stress worsens COPD outcomes by triggering exacerbations of coughing, wheezing, and breathlessness [20, 21]. Living with a chronic illness also creates ongoing emotional strain, leading to social isolation and financial hardship [22, 23, 24]. This chronic stress may further weaken immune responses and amplify physical symptoms [25, 26]. Despite its clinical importance, stress in COPD patients often goes underrecognized due to time constraints, lack of training, or stigma surrounding mental health [22, 27, 28].

Breathing exercises are an important part of non‐pharmacological management in COPD, as they improve respiratory efficiency and promote relaxation [29, 30]. Practices such as incentive spirometry, balloon blowing, and pursed‐lip breathing can reduce stress by modulating the autonomic nervous system, lowering sympathetic activation while enhancing parasympathetic activity [31, 32, 33]. Incentive spirometry in particular offers visual feedback to patients, encouraging deep, sustained inspiration and strengthening inspiratory muscles [34, 35].

Although incentive spirometry is widely prescribed for preventing atelectasis in postsurgical patients, its potential to reduce psychological stress in COPD has not been extensively investigated [35, 36]. This study was therefore designed to determine whether incentive spirometry exercise could reduce perceived stress in COPD patients, offering a simple, inexpensive, and patient‐centered strategy to improve mental health alongside respiratory rehabilitation.

## 2 Methodology

This pretest‐posttest quasi‐experimental study was conducted at the Department of Internal Medicine, District Headquarter Teaching Hospital, Rawalpindi, over a period of 12 months from December 2021 to December 2022, following approval of the synopsis. A total of 100 diagnosed COPD patients were included using convenient purposive sampling after obtaining written informed consent. Participants were divided into two groups: a control group that received routine medical management, and an intervention group that performed incentive spirometry exercise in addition to routine medicines for 8 weeks using convenient purposive sampling.

The sample size of 100 cases was measured from the formula below by taking the confidence level 95% and power of study 80%. A previous study found that red cell distribution width in COPD patients was 15% ± 2.3% [37]. Assuming a 10% reduction in red cell distribution width by breathing exercise, the sample size was calculated as 100, with 50 participants in each group.

$$n=\left[2\sigma^{2}{\left(Z_{1-\alpha /2}+Z_{1-\beta }\right)}^{2}\right]/{\left({µ}_{1}-{µ}_{2}\right)}^{2}$$

Eligible participants were patients with COPD, defined by the presence of chronic productive cough, airflow obstruction, progressively increasing breathlessness, hemoptysis or morning headache, radiological evidence of hyperinflated lungs, a flattened diaphragm, diminished vascular markings, and FEV_1_/FVC less than 70%. Patients were included if they had grade 0 to grade 2 dyspnea on the modified Medical Research Council scale and were willing to participate. Those with oral lesions, grade 3 to 4 dyspnea, or who were already practicing breathing exercises were excluded.

The primary outcome for this study was the perceived stress score. Stress levels were assessed at baseline, at 4 weeks, and after 8 weeks using the 10‐item Perceived Stress Scale questionnaire, which was completed by all participants in both groups.

Participants in the intervention group performed incentive spirometry using a flow‐oriented Tri‐Ball device. They were instructed to sit upright, exhale normally, and then take a deep maximum inspiration through the mouthpiece of the spirometer, holding their breath for as long as possible. The uplifted balls provided visual feedback of their inspiratory effort. Each participant was asked to complete 5–10 breaths per session, at least once every hour while awake with a minimum of 9 sessions in a day. Training began on the first day of hospitalization and continued for 8 weeks. After hospital discharge, the adherence to incentive spirometry was monitored using a paper based log which was provided to the participants on their discharge. The log contained hourly boxes. The participants were instructed to tick hourly log box each time they completed the prescribed breathing exercises. Adherence was assessed via telephone calls weekly.

Data was entered into SPSS version 23. Quantitative data such as age and perceived stress score were presented as mean and standard deviation, while categorical data like gender were presented as frequency and percentage. The independent sample *t*‐test compared perceived stress scores between the two groups. The paired sample *t*‐test was used to compare stress scores within groups. Prior to parametric testing, the normality of PSS‐10 score distributions was verified using the Shapiro–Wilk test, which confirmed approximate normality in both groups.

## 3 Results

A total of 100 participants were included in the study. The mean age of the study participants was 53.88 ± 10.83 years. Out of 100 participants, 89% (89) were male and 11% (11) were female. The mean BMI was 24.12 ± 3.51 kg/m². The basic characteristics of the study participants are presented in Table 1.

**Table 1:** Basic characteristics of study participants (*N* = 100).

Variable	Control group	Intervention group	*p* value
Number of participants (*n*)	50	50	
Male *n* (%)	44 (88)	45 (90)	
Female *n* (%)	6 (12)	5 (10)	
Age (years)	54.2 ± 11.1	53.6 ± 10.6	0.687
BMI (kg/m²)	24.3 ± 3.6	23.9 ± 3.4	0.101
Perceived stress score	17.66 ± 7.46 (95% CI: 15.54–19.78)	19.10 ± 7.45 (95% CI: 16.98–21.21)	0.33
FEV1/FVC (%)	71.97 ± 5.77 (95% CI: 70.23–73.69)	71.02 ± 5.90 (95% CI: 69.27–72.78)	0.997

Perceived stress scores of all study participants were measured at baseline (Day 0). Fifty participants were allocated to the control group and 50 to the exercise group. Independent sample *t*‐test analysis showed no significant difference in perceived stress score between the two groups at baseline (Table 1).

During the study, five participants in the control group dropped out. Whereas in the exercise group one participant refused to continue and two participants were not compliant to IS exercise, so a total of three participants in the exercise group dropped out. The dropout of participants in both groups results in an overall completion rate of 92% (92). At the end of 4 week intervention, the perceived stress score of the control group was 17.38 ± 7.01 while the perceived stress score of the exercise group was 17.36 ± 5.61. At the end of 4 week intervention, the FEV1/FVC (%) of control group was 71.77 ± 5.68 while the FEV1/FVC (%) of exercise group was 73.13 ± 5.58.

At the end of the 8‐week intervention, the exercise group demonstrated a significant reduction in perceived stress score compared to the control group (Table 2). Within‐group analysis showed no significant change in stress score for the control group. In contrast, a significant reduction in perceived stress score was observed in the exercise group from baseline to 8 weeks (Table 3).

**Table 2:** Comparison of perceived stress scores and FEV1/FVC (%) between control and exercise groups at baseline and after 8 weeks.

Variable	Control (baseline) mean ± SD 95% CI	Exercise (baseline) mean ± SD 95% CI	*p* value	Control (8 weeks) mean ± SD 95% CI	Exercise (8 weeks) mean ± SD 95% CI	*p* value	Effect size (Hedges' *g*)
Perceived stress score	17.66 ± 7.46 (95% CI: 15.54–19.78)	19.10 ± 7.45 (95% CI: 16.98–21.21)	0.336	18.10 ± 7.57 (95% CI: 15.83–20.38)	14.51 ± 6.47 (95% CI: 12.66–16.41)	0.016*	0.510718 (medium)
FEV1/FVC (%)	71.97 ± 5.77 (95% CI: 70.23–73.69)	71.02 ± 5.90 (95% CI: 69.27–72.78)	0.997	71.83 ± 5.65 (95% CI: 70.14–73.53)	75.58 ± 5.08 (95% CI: 74.09–77.08)	0.001	

* means *p* < = 0.05 (significant).

**Table 3:** Within‐group changes in perceived stress score from baseline to 8 weeks.

Variable	Baseline mean ± SD	8 Weeks mean ± SD	*p* value
Control group (*n* = 45)	17.31 ± 7.68 (95% CI: 15.54–19.78)	18.11 ± 7.57 (95% CI: 15.83–20.38)	0.159
Exercise group (*n* = 47)	19.11 ± 7.25 (95% CI: 16.98–21.21)	14.51 ± 6.47 (95% CI: 12.66–16.41)	0.000*

* means *p* < = 0.05 (significant).

Adherence to the prescribed incentive spirometry protocol was monitored via paper‐based hourly logs and weekly telephone calls. All 47 participants who completed the exercise intervention maintained the minimum prescribed dose of 9 sessions per day throughout the 8‐week period. Adherence ranged from 9 to 12 or more sessions daily across participants, with no completer falling below the minimum threshold. The two participants who failed to meet this threshold were identified through monitoring and were classified as dropouts.

The initial sample size calculation was based on preliminary estimates using RDW as a surrogate marker. However, to appropriately justify the adequacy of our final sample, we conducted a post‐hoc power analysis based on our primary outcome (perceived stress score) using GPower 3.1. With the observed effect size of Hedges' *g* = 0.51 (medium) and achieved sample size of *n* = 92, the study attained approximately 69% statistical power at an alpha level of 0.05 (two‐tailed, independent samples *t*‐test). While this power is below the conventional threshold of 80%, it remains adequate to detect the observed medium effect size for our primary stress outcome. The achieved sample size was sufficient to detect the clinically meaningful reduction in perceived stress scores demonstrated in our results (*p* = 0.016), with the minimum clinically important difference (MCID) for the PSS‐10 ranging from 2.19 to 2.66 points, confirming that dropout did not compromise the study's ability to detect significant effects.

## 4 Discussion

Chronic obstructive pulmonary disease patients often experience breathlessness and reduced exercise capacity, leading to reduced quality of life and increased stress levels. In fact, a meta‐analysis reported pooled prevalence rates of anxiety and depression in COPD populations of about 36% and 25%, respectively, emphasizing that psychological comorbidities are not uncommon in this group [20]. There is growing evidence that people with COPD may also struggle with a number of psychological issues, in addition to the severity of their physical symptoms, which can have a negative impact on their quality of life. Patients diagnosed with COPD are widely believed to suffer from depressed mood, anxiety, and a lower quality of life [38].

Perceived stress refers to an individual's impression of their stress and the degree to which they are having trouble adjusting to it. Chronic stress has been associated with negative health outcomes, including exacerbation of COPD symptoms [39]; this worsening of COPD symptoms creates a positive feedback loop on stress levels. In our study, we observed that incentive spirometry intervention was associated with a statistically significant reduction in perceived stress scores among COPD patients (*p* < 0.05).

Non‐pharmacological interventions, which include breathing exercises, smoking cessation, physical activity, and vaccination form an integral component of comprehensive COPD management. Among non‐pharmacological interventions, breathing exercises have been used previously to reduce exacerbations in COPD patients. These interventions play their role by mitigating inflammation‐driven COPD progression. Pulmonary rehabilitation and exercises reduce the pro‐inflammatory cytokines (TNF‐α, IL‐6) and oxidative stress. Breathing exercise also improves exercise capacity and clinical outcomes of COPD patients [40]. Breathing exercises such as diaphragmatic breathing, slow‐paced breathing, and alternate‐nostril breathing are increasingly recognized for their ability to modulate autonomic tone and reduce stress. Effective breathing practices enhance parasympathetic activity, thereby counterbalancing the heightened sympathetic tone commonly observed in stress and anxiety [32]. Moreover, a randomized clinical trial in hospitalized COPD patients showed that controlled breathing techniques significantly reduced anxiety and depression scores (HADS) over 8 weeks [41].

According to the findings of a study that was carried out by Sadiq et al., blowing a balloon can enhance pulmonary function tests and lower stress levels in medical students [42]. Although our breathing exercise also reduced the perceived stress level in incentive spirometer exercise group participants, we conducted our study on COPD patients instead of stressed medical students and used incentive spirometry exercise instead of balloons used by Sadiq et al. Another study conducted by Sadiq et al. showed that improvement of pulmonary function tests lead to improved heart rate variability and reduced stress in medical students [43]. A similar effect is assumed; however, further investigation is necessary to confirm this result.

Stress is characterized physiologically as sympathovagal imbalance and sympathetic dominance [44]. The act of engaging in breathing exercises through incentive spirometry results in increased sustained inflation of the lungs and stimulation of slow‐adapting pulmonary stretch receptors (SARs). Increased afferent discharge from SARs can increase vagal outflow to the heart, supporting parasympathetic dominance and decreased heart rate, which can all lead to lessening the bodily stress response [32, 45]. This inhibitory neural reflex mechanism is one reasonable pathway for breathing interventions to lessen perceived stress. Indeed, breathing interventions have been, and still are, often characterized as having their effects via cardiorespiratory reflex pathways [32, 45].

Moreover, breathing‐based interventions showed even wider benefits in COPD. A systematic review of 73 randomized controlled trials (*n* = 5479) found that breathing exercises (i.e., diaphragmatic, and pursed‐lip breathing) decreased dyspnea (mean difference −0.40 mMRC, 95% CI −0.70 to −0.11) and improved health‐related quality of life, as compared to usual care [46]. Another systematic review found that breathing exercises can improve exercise endurance, respiratory muscle strength, and quality of life in COPD populations, although methodological limitations exist in many trials [47].

In terms of respiratory mechanics, including incentive spirometry (IS) with inspiratory muscle training (IMT) produces important improvements in lung function. For example, in a study of COPD patients undergoing lung resection, the IS + IMT group attained postoperative forced expiratory volume (FEV₁) values that were 570 mL higher than predicted in lobectomy cases (compared to the control group, which underestimated by ~70 mL) and 680 mL higher in pneumonectomy cases [48].

While that was in the surgical context, it is endorsing that IS might enhance functional respiratory improvements when utilized properly. Another study implementing threshold IMT on its own in stable COPD, generated an increase in FEV₁ of ~0.08 L (95% CI 0.02–0.13 L) and a significant difference in inspiratory muscle strength and distance walked (~34 m) while having no clear change in quality of life [49, 50]. Our findings align with recent evidence demonstrating the effectiveness of structured inspiratory muscle training in COPD. A randomized controlled trial by Mohammed et al. compared threshold inspiratory muscle training with diaphragmatic and pursed‐lip breathing in occupational COPD patients, demonstrating that structured inspiratory muscle training produces meaningful respiratory improvements [51]. These findings support incentive spirometry as an evidence‐based, feasible non‐pharmacological intervention for respiratory and psychological benefits in COPD populations.

A person who suffers from stress has sympathovagal imbalances, with a dominance of sympathetic activity. When a person performs exercise via incentive spirometer his lungs become overstretched which causes an increase in excitatory signal discharge from slow adapting pulmonary stretch receptors to preganglionic cardiac vagal nerve. This increase in discharge results in an increase in vagal nerve activity. The increased vagal nerve activity shifts autonomic activity towards parasympathetic dominance. This mechanism explains how breathing exercises, including incentive spirometry results in a reduction of stress [43]. Beyond statistical significance, our results demonstrate clinical meaningfulness. The minimum clinically important difference (MCID) for the PSS‐10 ranges from 2.19 to 2.66 points [52]. The observed mean reduction in the exercise group was 4.60 points, that is, stress level of our exercise group was reduced from 19.11 points to 14.51 points, which substantially exceeded this MCID threshold, indicating that the stress reduction achieved by incentive spirometry represents a clinically meaningful benefit for COPD patients.

This study has several important limitations that should be considered when interpreting the findings. First, the quasi‐experimental design without true randomization limits causal inference, moreover we have conducted our study at a single center, potentially limiting generalizability. Our study sample was predominantly male (89%), which may not represent the broader COPD population and limits the generalizability of findings to female patients. Moreover, the post‐hoc power analysis revealed that the study achieved only 69% statistical power (GPower 3.1, effect size *g* = 0.51, *n* = 92, *α* = 0.05), falling below the conventional 80% threshold. This underpowering increases the risk of Type II error and means that some real effects may not have been detected; future studies with larger samples are warranted. The follow‐up period was relatively short (8 weeks), and long‐term sustainability of stress reduction benefits remains unknown. Future studies with extended follow‐up periods (12–24 weeks) are needed to assess the durability of effects.

Our primary outcome relied solely on a self‐reported subjective measure (PSS‐10) without objective validation through physiological markers such as cortisol levels, heart rate variability, or salivary biomarkers that could corroborate psychological stress reduction. In future, the effect of IS can be studied by observing its effect on biomarkers in COPD patients.

## 5 Conclusion

The present study shows that incentive spirometry significantly reduced perceived stress in COPD patients. These findings indicate that while IS improves respiratory mechanics, there is potential for improving psychology through its means of deep breathing, which restores sympathovagal balance. With chronic stress being tied to worse outcomes in COPD, the decrease in stress score is important, highlighting IS as a low‐cost and simple addition to stable COPD care. Future research in larger cohorts during a lengthier follow‐up period is indicated to substantiate our findings and the extent to which incentive spirometry remains an effective method for decreasing stress.

## Author Contributions

**Noor Nasir Rajpoot:** conceptualization, data curation, formal analysis, investigation, methodology, project administration, resources, software, visualization, and writing – original draft. **Noman Sadiq:** conceptualization, data curation, formal analysis, investigation, methodology, project administration, resources, software, supervision, validation, visualization, and writing – original draft preparation. **Quang Dai La:** formal analysis, writing – original draft, writing – review and editing. **Tahira Sadiq:** conceptualization, investigation, writing – original draft, methodology, validation, visualization, software, project administration, formal analysis, data curation, supervision, and resources. **Eric Teng:** writing – original draft, writing – review and editing. **Han B. La:** writing – original draft, writing – review and editing. **David M. Lo:** writing – original draft, writing – review and editing, validation.

## Funding

The authors have nothing to report.

## Ethics Statement

This research was approved by the Institutional Research Forum and the Research and Ethical Committee of Rawalpindi Medical University on January 22, 2021 under ref. no.15/IREF/RMU/2021.

## Consent

All data was collected after obtaining written informed consent.

## Conflicts of Interest

The authors declare no conflicts of interest.

## Transparency Statement

The corresponding author, David M. Lo, affirms that this manuscript is an honest, accurate, and transparent account of the study being reported; that no important aspects of the study have been omitted; and that any discrepancies from the study as planned (and, if relevant, registered) have been explained.

## Data Availability

The data that support the findings of this study are available on request from the corresponding author. The data are not publicly available due to privacy or ethical restrictions.
